# Advances in Hydrophilic Drug Delivery: Encapsulation of Biotin in Alginate Microparticles

**DOI:** 10.3390/pharmaceutics17091117

**Published:** 2025-08-27

**Authors:** Iria Naveira-Souto, Elisabet Rosell-Vives, Eloy Pena-Rodríguez, Francisco Fernandez-Campos, Maria Lajarin-Reinares

**Affiliations:** 1 Department of Biochemistry and Physiology, School of Pharmacy and Food Sciences, University of Barcelona, 08028 Barcelona, Spain; inaveira@reigjofre.com; 2 Pharmaceutical Innovation, Reig Jofre Laboratories, 08970 Barcelona, Spain; erosell@reigjofre.com (E.R.-V.); eloy.penarodriguez@microcaps.ch (E.P.-R.); francisco.fernandez@labiana.com (F.F.-C.)

**Keywords:** alginate, chitosan, drug release, biotin, microparticles, ionic reticulation, design of experiments

## Abstract

**Background:** The encapsulation of hydrophilic drugs within microparticles has gained significant interest in drug delivery systems due to their potential to improve stability, bioavailability, and controlled release of therapeutic agents. Biotin, a water-soluble vitamin, presents challenges such as rapid degradation and limited membrane permeability, which constrain its therapeutic effectiveness. **Objectives:** This study aims to develop and characterize biotin-loaded microparticles formulated with alginate, Eudragit^®^ E100, and CaCl_2_, and to evaluate their characterization and potential applications. **Methods:** The microparticles were produced using the external ionic gelation method, where alginate and CaCl_2_ solutions were mixed under probe sonication. Eudragit^®^ E100 was added as a complexing agent. The optimized formulation was used to encapsulate biotin, and various experimental variables were screened to study their influence on the properties of the microparticles. **Results:** Biotin was encapsulated in alginate microparticles (size: 634 nm; polydispersity index: 0.26; zeta potential: −45 mV) with an encapsulation efficiency of 90.5%. In vitro release studies using vertical diffusion Franz cells demonstrated a controlled release profile following the Weibull kinetic model. **Conclusions:** Encapsulation techniques offer a promising approach to overcome the limitations of hydrophilic drug delivery. The biotin-loaded microparticles developed in this study have potential applications in both topical and oral formulations, providing controlled release and improved therapeutic efficacy, and illustrate the broader applicability of polymeric encapsulation systems for improving the delivery of labile, hydrophilic bioactives.

## 1. Introduction

The development of effective delivery strategies for hydrophilic drugs has emerged as a critical area of pharmaceutical research, driven by the increasing interest in both macromolecular therapeutics—such as nucleic acids, peptides, and proteins—and small hydrophilic molecules for the treatment of cancer, infectious, and inflammatory diseases [1]. Despite their therapeutic potential, hydrophilic drugs frequently suffer from limited membrane permeability, rapid degradation, and short half-lives, which hinder their clinical efficacy and bioavailability [2,3].

Microencapsulation presents a highly effective strategy to overcome these limitations by enhancing the delivery of hydrophilic compounds such as biotin. Microparticles (MPs)—spherical structures typically ranging from 1 to 1000 µm in diameter—can protect sensitive bioactives from adverse environmental conditions (e.g., pH fluctuations, oxidation, temperature), enhance chemical and enzymatic stability, and allow for controlled and targeted release. These polymeric delivery systems can be incorporated into a wide array of pharmaceutical dosage forms, including solids (e.g., tablets, capsules), semisolids (e.g., gels, creams), and liquids (e.g., suspensions), making them highly adaptable to different therapeutic needs [2].

**Figure 1:**
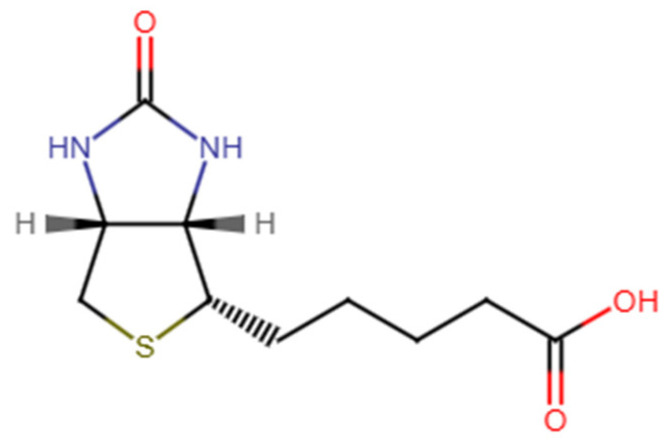
Chemical structure of d-(+)-biotin.

Biotin, also known as vitamin B7 [4], is a water-soluble compound that functions as a cofactor in various metabolic pathways, essential for maintaining healthy skin, hair, and nails, as well as promoting cellular growth and division [5,6]. Chemically identified as cis-hexahydro-2-oxo-1H-thieno [3,4-d] imidazole-4-pentanoic acid, biotin consists of two heterocyclic rings (Figure 1) [7]. With a pKa of 4.5, the vitamin remains stable between pH 4 and 9 and is insoluble in organic solvents, with limited water solubility (20 mg/100 mL) [8]. Since mammals are unable to synthesize biotin endogenously and rely on dietary intake and bacterial biosynthesis in the gut to meet their needs, effective supplementation methods are essential [8,9,10]. The physiological importance of biotin is primarily highlighted by the pathological effects that occur with its deficiency, which can lead to a variety of symptoms affecting the nervous system, skin, and respiratory system [6,10]. However, its hydrophilic nature poses delivery challenges, where precise dosing and controlled release are paramount for maximizing efficacy while minimizing side effects [11,12,13].

**Figure 2:**
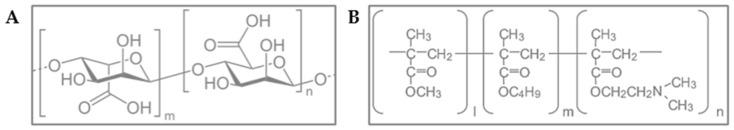
(**A**). Chemical structure of alginate, where m and n correspond to the number of repeated units of α-D-glucuronic acid and β-D-mannuronic acid, respectively, (**B**) Chemical structure of Eudragit^®^ E100, where l, m, and n correspond to the number of repeated units of methyl metacrylate, butyl metacrylate, and dimethylaminoethyl methacrylate, respectively.

This study presents the innovative fabrication of alginate–Eudragit^®^ E100 microparticles for the encapsulation of biotin using the external ionic gelation technique [14]. Alginate, a naturally derived anionic polymer obtained from brown algae consisting of α-L-guluronic acid and β-D-mannuronic acid residues linearly linked by 1,4-glycosidic bonds (Figure 2A), is widely recognized for its biocompatibility, biodegradability, mucoadhesiveness, and non-immunogenicity, making it a suitable candidate for biomedical and pharmaceutical applications [15]. Alginates exhibit significant variability in composition and sequence; the specific alginate used in this study had a molecular weight of 200 kDa [14,16]. Moreover, alginate’s compatibility with other polymers, ease of formulation, and cost-effectiveness make it an ideal material for developing both topical and oral drug delivery systems [17].

**Figure 3:**
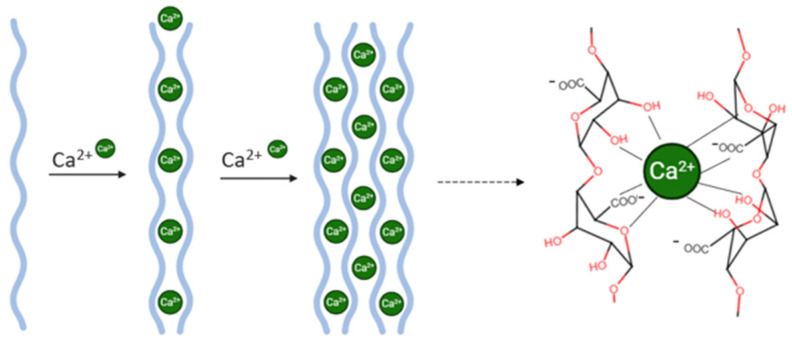
Ionic gelation process with egg-box junction of calcium and alginate.

A key advantage of alginate is its ability to undergo gelation under mild conditions in the presence of divalent cations—such as calcium (Ca^2+^)—avoiding the use of harsh solvents or high temperatures, thus preserving the integrity of sensitive bioactives like biotin (Figure 3) [14,15]. In this study, CaCl_2_ was selected as the cross-linking agent due to its high aqueous solubility and ability to diffuse efficiently into the alginate matrix, promoting the formation of a stable hydrogel network [16].

Alginate-based microparticles have been extensively studied for the encapsulation of hydrophilic drugs due to their protective and release-modulating properties. Previous research has demonstrated their effectiveness in the delivery of compounds such as insulin, vitamin C, and antibiotics, in both oral and topical formulations [18,19,20]. However, the application of alginate-based systems for biotin delivery remains relatively unexplored, and studies evaluating its combination with cationic polymers such as Eudragit^®^ E100 are currently lacking. This highlights the need for novel delivery platforms capable of improving the stability and bioavailability of biotin under physiological conditions.

To enhance the structural and functional properties of the microparticles, Eudragit^®^ E100 was incorporated as a cationic complexing agent. This copolymer—composed of dimethylaminoethyl methacrylate, butyl methacrylate, and methyl methacrylate—is widely recognized for its stability, biocompatibility, and high skin tolerance (Figure 2B). It is used extensively in pharmaceutical formulations, including use as an excipient in solid dosage forms (to mask flavors and odors and provide moisture protection), as a coating agent, and for controlling drug release in various formulations [21]. Its electrostatic interaction with the negatively charged glucuronic acid residues of alginate reinforces the polymeric matrix, leading to the formation of more stable and robust microparticles [22]. This interaction enhances mechanical strength and encapsulation efficiency, introducing a novel strategy beyond conventional alginate–calcium systems, which are more susceptible to ionic exchange and environmental instability. In contrast, the complexation with Eudragit^®^ E100 provides enhanced structural integrity, pH responsiveness, and potential for more precise control over the release profile of the encapsulated agents. Accordingly, microparticles have been identified as an effective strategy for the delivery of hydrophilic substances. In recent years, statistical designs have seen widespread application in the investigation of pharmaceutical processes and formulations, enabling researchers to assess the influence of process parameters or compositional variables on final product characteristics. Typically, factorial or response surface designs serve as initial approaches in experimental design, allowing systematic variation of factors at multiple levels. The goal was to obtain a formulation with the optimal ratio of these polymers (alginate and Eudragit^®^ E100), designed as a versatile platform for the encapsulation of hydrophilic compounds, with biotin selected as a model compound for proof-of-concept studies and system characterization, with the optimization process guided by a design of experiment [23,24,25].

By combining the complementary properties of alginate and Eudragit^®^ E100, the proposed microparticulate system enables efficient biotin encapsulation, offering protection against degradation and supporting controlled release. This novel delivery approach holds promise for improving the therapeutic efficacy of hydrophilic vitamins and can be adapted for a wide range of pharmaceutical and nutraceutical applications.

Although biotin conjugates with alginate have previously been explored for biosensor construction [11], to the best of our knowledge, this is the first report of biotin encapsulation in alginate-based microparticles for controlled drug delivery applications. This work aims to address the need for more stable and bioavailable formulations of biotin, a clinically relevant vitamin involved in metabolic, dermatological, and neurological processes. The development of microparticles for the protection of the model compound biotin, illustrating the capacity of the platform to enhance stability and control the release of hydrophilic molecules, could offer new therapeutic opportunities, particularly for populations with absorption issues or increased vitamin requirements. Beyond biotin, this strategy could serve as a versatile platform for enhancing the delivery of other labile or poorly absorbed hydrophilic compounds, particularly in formulations where stability and targeted release are essential.

## 2. Materials and Methods

### 2.1. Materials

Sodium alginate (MW (g/mol) > 200,000, Carbo Erba Reagents, Val de Reuil Cedex, France), Basic Butylated Methacrylate Copolymer (Eudragit^®^ E100) (Evonik, Darmstadt, Germany), calcium chloride dihydrate (Scharlab, S.L., Sentmenat, Spain) and purified water (in house) were used to prepare microparticles. Biotin (Kingland Trading Company, Iowa, IA, USA) was the active ingredient used to formulate the MP. Sodium perchloride monohydrate (Scharlab, S.L., Sentmenat, Spain), phosphoric acid (Scharlab, S.L., Sentmenat, Spain), and acetonitrile (Scharlab, S.L., Sentmenat, Spain) were used to perform the different analyses.

### 2.2. Synthesis of Alginate-MP and Biotin-Loaded Alginate-MP

Three solutions were prepared at different concentrations of alginate (0.1–0.4% *w*/*w*), CaCl_2_ (0.01–0.04% *w*/*w*), and Eudragit^®^ E100 (0.01–0.2% *w*/*w*). The concentration range was selected based on a previous unpublished formulation study. The alginate and CaCl_2_ solutions were made in purified water. Eudragit^®^ E100 was dissolved in 0.1 N hydrochloric acid, since it is a copolymer that only dissolves at acidic pH (<5) [22]. As the initial phase, the mixture between the CaCl_2_ solution and the alginate was carried out at 500 rpm for 5 min in a beaker. Afterwards, in the second stage, the Eudragit^®^ E100 was added, and all batches were sonicated (2000–8000 Ws). The gelation process was carried out under probe sonication using a UP400st ultrasonic device (Hielscher Ultrasonics, Teltow, Germany) to reduce the size of the particles. The whole process was performed at room temperature.

The optimized formulation was used to encapsulate biotin. Biotin was incorporated at 0.05% *w*/*w* of the final formulation by dissolving it in the alginate phase containing 2.35% *w*/*w* of NaOH 0.1 N. Biotin-loaded alginate-MPs were then formed following the same procedure as the non-loaded alginate-MPs. After particle formation, the pH was adjusted to 4 with HCl 0.1 N.

### 2.3. Development of Alginate Microparticles

Preliminary screening trials were conducted. Twelve placebo batches (*n* = 12) were prepared to evaluate the influence of formulation and process parameters—including alginate, Eudragit^®^ E100, and CaCl_2_ concentrations, sonication energy (Ws), and sonication amplitude (%)—on key physicochemical properties: zeta potential (Z-potential) (mV), hydrodynamic diameter (Z-average, nm), and polydispersity index (PDI) (Table 1). A Plackett–Burman design was employed to identify significant main effects of these independent variables (α = 0.05). Model selection was based on R^2^ and adjusted R^2^. To minimize potential bias from external factors, the formulation production order was randomized. The selection of polymer concentration and sonication energy ranges was guided by previous published work [14,26,27,28] and initial formulation tests. Only those ranges that demonstrated favorable results from a formulation perspective—such as homogeneous appearance, stable Z-potential, and a Z-average less than 2000 nm—were chosen.

Statistical analysis of the variables studied was carried out using Minitab 17 statistical software (Minitab, Inc., 2010, State College, PA, USA) to obtain mathematical equations for each model. Subsequently, each model was optimized.

Once the screening study was completed, only the significant variables of the model were used to optimize microparticles’ Z-average and PDI (Z-potential was excluded from the analysis, as explained in the Section 3 based on a central composite design. For this, 6 central points, 8 cube points, and 6 axial points were selected. A default α value of 1.4 was used to determine the axial points. A significance level (α) of 0.05 was established. The choice of the model was made based on the values of R^2^ and adjusted R^2^.

After the optimization, the final mathematical model obtained was used to produce microparticles. To evaluate the predictive capacity of the model, the properties of the optimized microparticles were compared with those predicted by the model, and the bias was calculated (Equation (1)).

(1) $$\%\mathrm{Bias}=\frac{\left|\;Ref-Z1\right|\;}{Ref}\;\times 100$$

where *Ref* is the theoretical reference value of the model, and *Z*_1_ is the mean of the experimental value obtained.

### 2.4. Microparticles Physicochemical Characterization

#### 2.4.1. Hydrodynamic Diameter, Polydispersity Index, and Z-Potential

The Z-potential (Z-Pot), hydrodynamic diameter (Z-Ave), and polydispersity index (PDI) were studied for alginate particles through dynamic light scattering (DLS) using a Malvern Zetasizer Nano ZS (Malvern Panalytical, Malvern, UK) (*n* = 3).

Three measurements were performed on each sample with 15 readings per measurement. The results obtained were considered correct when the autocorrelation function was between 0.6 and 1. PDI and the Z-Ave size were carried out in 10 mm × 10 mm × 45 mm polystyrene cuvettes. In the case of Z-Pot, DST1070 cells were used, which consist of two gold-covered copper electrodes (Malvern Panalytical, Worcestershire, UK).

#### 2.4.2. Transmission Electron Microscopy

The final placebo alginate-MPs and biotin-loaded alginate-MP morphology and structural features were studied using transmission electron microscopy (TEM) (FEI, Eindhoven, The Netherlands). The sample was adsorbed onto a carbon-coated copper grid for 1 min and then washed with milli-Q^®^ water (Fisher Scientific S.L, Alcobendas, Madrid) for 1 min. It was negatively stained by floating the grid on a drop of 2% phosphotungstic acid for 1 min. The excess of liquid was blotted manually from the edge of the grids. The sample was observed in a Tecnai Spirit TEM (FEI, Eindhoven, The Netherlands) equipped with a tungsten cathode. Images were acquired at 120 kV with a 1kx1k Megaview III CCD camera.

### 2.5. High-Performance Liquid Chromatography (HPLC) Analysis, Encapsulation Efficiency, and Biotin Content Determination

The encapsulation efficiency (%*EE*) of biotin in the particles was determined indirectly following Equation (2), where *W_T_* denotes the total content of biotin in the formulation and *W_NE_* denotes the biotin obtained in the filtrate (not encapsulated) after centrifugation using an Amicon Ultra device (Merck Millipore, Barcelona, Spain) with a membrane cutoff of 100 KDa at 2200× *g* for 30 min (*n* = 3).

(2) $$\%EE=\frac{W_{T}-W_{NE}}{W_{T}}\times 100$$

The biotin content was quantified using HPLC (Waters 2695, Cerdanyola del Vallès, Spain) with a photodiode array detector (Waters 2996, Cerdanyola del Vallès, Spain), following a calibration curve (range: 20 to 0.1 µg/mL, r^2^ > 0.999). The column used was a C18 (4.6 mm × 150 mm) with a 3.5 µm particle size. The mobile phase was a gradient of an aqueous solution (mobile phase A) prepared with sodium perchloride monohydrate 0.1% (*w*/*v*) with concentrated phosphoric acid 0.1% (*v*/*v*) and acetonitrile (mobile phase B). The gradient was structured as shown in Table 2. The flow rate was 1.0 mL/min, and the injection volume was 20 µL. The sample and column temperature were set at 25 °C.

The biotin content in the final formulation (*w*/*w*) was calculated theoretically as the mass of biotin initially added to the formulation multiplied by the experimentally determined encapsulation efficiency (EE, determined by HPLC), divided by the total mass of the final dispersion. Drug loading on a dry particle basis was not determined, as particles were not lyophilized in this study.

### 2.6. In Vitro Release Tests

In vitro release of biotin-loaded alginate-MP was studied using Slide-A-Lyzer^®^ dialysis cassettes from Thermo Scientific (Cornellà, Spain) (0.5–3 mL, 3.500 MW cutoff). The selected MWCO of 3500 Da for the dialysis membrane was appropriate for biotin (MW ≈ 244 Da), allowing free diffusion of the released drug while retaining the microparticle matrix. This setup ensured that the observed release kinetics reflected the diffusion of biotin from the microparticles, rather than limitations imposed by the membrane itself. Prior to their use, the dialysis cassettes were soaked in phosphate buffer pH 7.2–7.4 (PBS) for 5 min as described in the instructions provided by the manufacturer. A dialysis cassette was then loaded with 3 mL of a solution of free biotin (in 2.4% NaOH 0.1N and milli-Q^®^ water) or biotin-loaded alginate-MPs (0.05%, *w*/*w*) and then immersed in 150 mL of PBS under continuous magnetic stirring at 300 rpm and room temperature. To ensure a direct comparison of release kinetics, the amount of encapsulated biotin present in the microparticle dispersion was equivalent to the amount of free biotin used (150 µg of biotin per cassette). This allowed for equivalent drug concentration across both formulations and minimized diffusion rate differences due to concentration gradients.

Aliquots of 300 µL were taken at certain times (0.25, 0.5, 1, 1.5, 2, 3, 22, 23, and 24 h) and injected into the HPLC with the method described in Section 2.6.

Kinetic modeling of the release data was studied with the DDSolver [23] (Microsoft Office 365, 2019)., an Excel add-in. Nonlinear representative mathematical models were fitted to the release curves to determine the best-fitting model: first-order, Higuchi, Korsmeyer–Peppas, and Weibull (Table 3). The selection of the best fit was based on the lowest AIC value.

### 2.7. Scale-Up of Alginate Particles

In order to reach higher production rates of alginate-MP, the first stage of the production process was scaled up.

**Figure 4:**
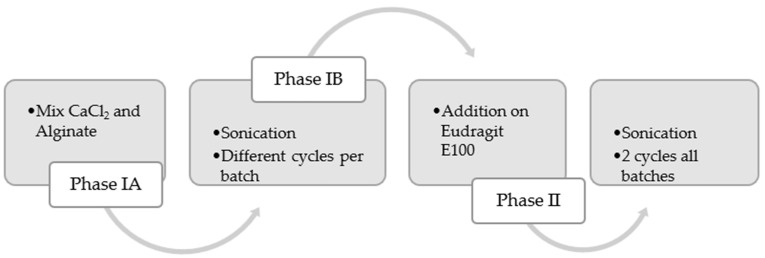
Process flow for scaling up alginate microparticles. The process is divided into two phases: Phase I involves mixing alginate and CaCl_2_ with sonication, and Phase II consists of adding Eudragit^®^ E100 with two additional sonication cycles.

**Figure 5:**
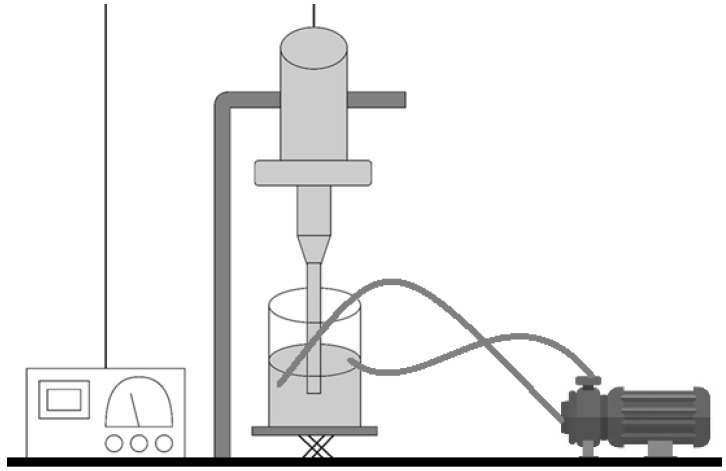
Schematic illustration of the device for manufacturing alginate microparticles on a large scale.

For the scale-up experiments, a UIP1000hdT ultrasonic device (Hielscher Ultrasonics, Teltow, Germany) (consisting of a transducer and ultrasonicator generator), a sonotrode (BS4d22; diameter 22 mm), and a flow cell reactor (FC100l1k-1S; Hielscher Ultrasonics, Teltow, Germany) were used. As the initial phase, the mixture between the CaCl_2_ solution and the alginate was carried out at 500 rpm in a beaker (Phase I, Figure 4). This vessel was connected to a peristaltic pump (Watson Marlow model 505S, Hielscher Ultrasonics, Teltow, Germany). At a flow rate of 2 L/min, it was recirculated to the flow reactor of the UIP1000hdT ultrasonicator. Considering the pump flow, volume, and number of specific cycles for each batch, the sonication time was determined (Phase IB, Figure 4). Afterwards, in the second stage, the Eudragit^®^ E100 was added, and all batches were sonicated for two more cycles (Phase II, Figure 4). The schematic illustration is shown in Figure 5.

During the process, a constant amplitude of 100% (220 µm) was used for continuous sonication. The batches were manufactured using the % composition determined by the particle’s optimization of Section 2.3. Every two sonication cycles, a sample was collected and characterized in accordance with Section 2.4.1. A sample was collected after the addition of Eudragit^®^ E100 and characterized in accordance with Section 2.4.1.

A central composite design with 13 runs, 5 central points, 4 cube points, and 4 axial points was selected to obtain the maximum information about scaling with the minimum number of batches. A default α value of 1.4 was used to determine the axial points using Minitab 17 statistical software. A significance level (α) of 0.05 was set to identify trends during the scale-up process. In this case, the effect of batch size and sonication cycles on the PDI and hydrodynamic diameter variables was determined (Table 4).

**Figure 6:**
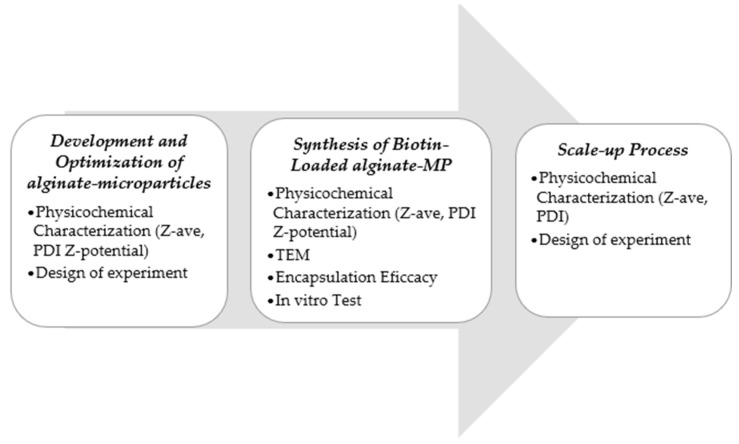
Schematic overview of the complete workflow, from microparticle synthesis to scale-up process.

An overview of the complete methodology employed in this investigation is provided in Figure 6.

## 3. Results and Discussion

### 3.1. Preparation and Statistical Design to Evaluate the Effect of Experimental Variables on the Physicochemical Characteristics of MPs

To evaluate the impact of the different excipients and manufacturing process on the physicochemical characteristics of the MPs (hydrodynamic diameter, polydispersity index and zeta potential), the values of the different variables were modified as follows: alginate concentration (0.1–0.3% *w*/*w*), CaCl_2_ concentration (0.01–0.03% *w*/*w*), Eudragit^®^ E100 concentration (0.05–0.2% *w*/*w*), sonication amplitude (20–60%), and total energy (2000–8000 Ws). As shown in Table 5, 12 formulation batches were produced, and their Z-Ave, PDI, and Z-Pot were subsequently measured.

A screening methodology was applied using Minitab^®^ software (with a significance level of α = 0.05 for the level terms) to evaluate the impact on the hydrodynamic diameter, polydispersity index, and zeta potential. Table 6 shows the fitted equations for the screening model with statistically significant variables. Model selection was based on the adjusted R^2^, which considers the different parameters in the nested models. The R^2^ values of the final equation indicate that it explains most of the experimental variability, mainly for the hydrodynamic size and zeta potential [26].

**Figure 7:**
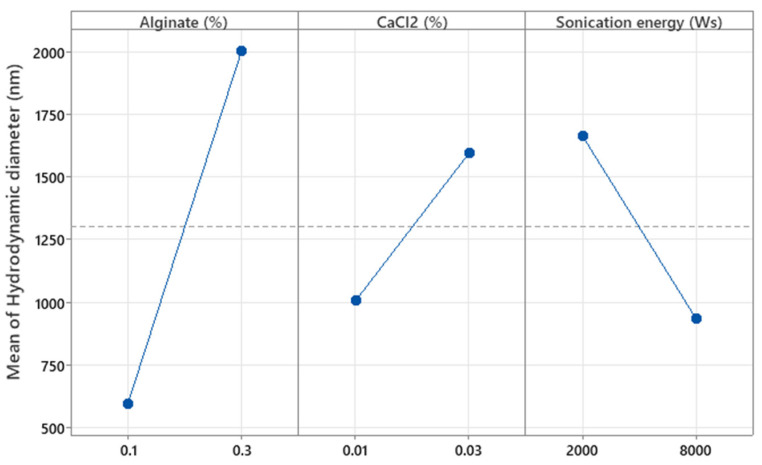
Plots of the main effect of alginate and CaCl_2_ concentration and sonication energy on the hydrodynamic diameter of the particles.

The factors X_1_, X_2,_ and X_3_ (concentrations of alginate and CaCl_2_ (%*w*/*w*) and the sonication energy (Ws), respectively) were significant with respect to Y_1_ (hydrodynamic diameter, *p* = 0.001, *p* = 0.044, and *p* = 0.020, respectively). Figure 7 shows that increasing the concentration of both alginates as well as CaCl_2_ results in a larger hydrodynamic diameter, as higher polymer and cross-linker concentrations lead to denser and more extensive molecular networks, promoting the formation of larger microparticles. This is because a higher amount of polymer provides more available chains for cross-linking, and a higher concentration of CaCl_2_ increases the availability of cross-linking ions, both contributing to the formation of a more compact and voluminous network structure [17]. In contrast, increasing the sonication energy applied to the MPs results in smaller particles, as the mechanical forces from sonication break down the polymeric network into smaller fragments. This occurs due to the cavitation phenomena generated during sonication, which induce high shear forces that fragment the larger polymer structures, thereby reducing particle size [27,28]. The effect of these factors was further analyzed in the surface response study.

**Figure 8:**
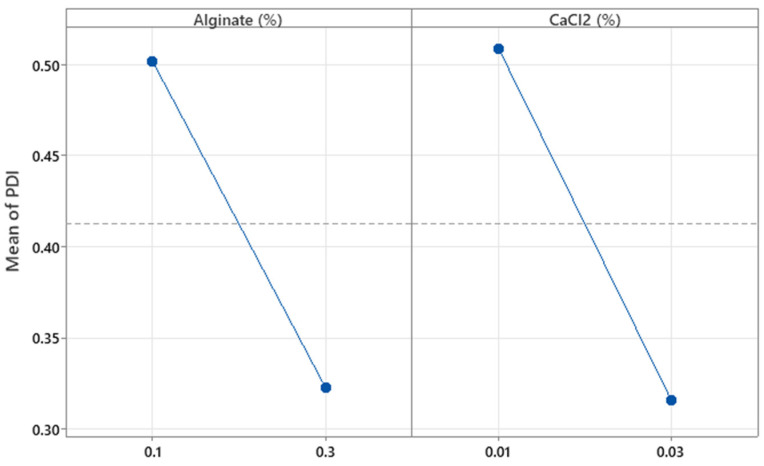
Plots of the main effect of alginate and CaCl_2_ concentration on the particle’s polydispersity index.

The concentrations of alginate and CaCl_2_ (%*w*/*w*) were not significant with respect to the PDI (*p* = 0.110 and *p* = 0.091, respectively). As shown in Figure 8, increasing the concentrations of alginate and CaCl_2_ tends to decrease the PDI values. However, these results do not fully explain the observed behavior, since the R^2^ value shows that only 52.24% of the variability in this parameter is explained by the independent variables. Further details on the effects of these factors were explored in the optimization experiments.

**Figure 9:**
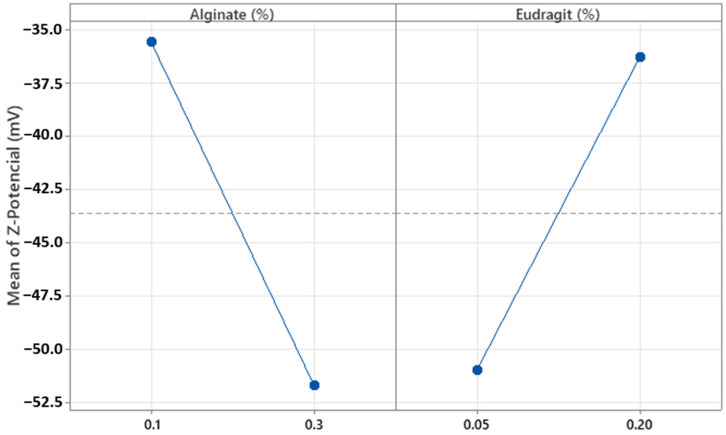
Plots of the main effect of alginate and Eudragit^®^ E100 concentration on the particle Z-average.

Regarding Z-Pot, the concentrations of alginate and Eudragit^®^ E100 (%*w*/*w*) were significant (*p* = 0.000 and *p* = 0.001, respectively). These results were consistent with the ionic nature of both components. The charges of polyelectrolytes with ionizable functional groups depend on the pH of the medium; in this case, the pH was around 5.1. Figure 9 shows that at this pH, increasing the concentration of alginate, which contains anionic groups, results in more negative Z-Pot values [29]. Conversely, increasing the concentration of Eudragit^®^ E100, which has positively charged groups, leads to more positive Z-Pot values. Additionally, all Z-Pot values obtained were negative, with most being below −30 mV (Table 5). When sufficiently high charges are present, the resulting MPs generate electrostatic repulsion forces promoting colloidal stability by preventing sedimentation and aggregation, thus enhancing the stability of the system [30]. These results show that the MPs are anionic, stable, and suitable for encapsulating hydrophilic drugs.

It should be noted that these measurements were carried out in the dispersion medium at pH 5.1, which corresponds to the medium in which the placebo microparticles were initially prepared and suspended. The aim at this stage was to evaluate the physiological properties of the carrier system itself. Subsequent formulation steps for specific routes of administration (e.g., oral or topical) would require further characterization at relevant physiological pH values.

### 3.2. Optimization of Alginate Microparticles

Once the main variables were obtained, the three factors (% alginate, % CaCl_2_, and sonication energy) were examined to optimize the Z-Ave and PDI of the formulation. The Z-potential was not optimized because during the screening study, all the values obtained were within the range considered stable <−25 mV [31]. As shown in Table 7, 20 formulation batches were produced, and the PDI and Z-Ave were characterized for each of them.

Surface response methodology was applied using Minitab software (with a significance level of α = 0.05 for the level terms) to analyze the effect of the tested experimental variables (Z-Ave and PDI). The fitted response surface model equations are shown in Table 8. Model selection was based on the adjusted R^2^, which considers the different parameters of the nested models. The R^2^ values (>0.7) of the final equation indicate that it explained most of the experimental variability [26].

**Figure 10:**
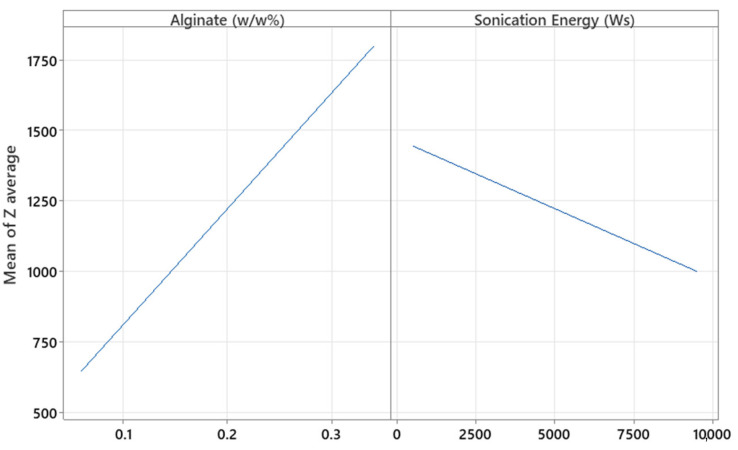
Plots of the main effect of alginate and sonication energy on the particle Z-average (nm).

The amount of alginate and sonication energy were significant with respect to Z-Ave (*p* = 0.000 and 0.037, respectively). By increasing the amount of polymer, the particle size increased, as expected (Figure 10). Although a higher polymer percentage allows for greater drug loading capacity or encapsulation of higher molecular weight substances [32], the primary reason for the increase in particle size is the rise in the viscosity of the medium at higher polymer concentrations, which promotes the formation of larger droplets during microparticle generation. Additionally, it was observed that CaCl_2_ concentration did not significantly affect particle size in this optimization study, in contrast to what was initially detected during the screening phase. In the screening study, where the CaCl_2_ concentration ranged from 0.01 to 0.03%, the *p*-value was 0.044, indicating a slight but statistically significant effect. However, in the optimization phase, the tested range was broader (0.006 to 0.034%) and the *p*-value was not significant, suggesting that within this expanded range, the effect of CaCl_2_ on particle size became negligible. In contrast, as more sonication energy was applied to the formulation, lower particle sizes were obtained. This effect could be explained by greater cavitation energy, leading to the breakup of larger particles into smaller ones [33]. Coefficient X_1_ is higher than X_2_, as shown in Table 8, indicating that polymer concentration had a greater influence on particle size than sonication energy.

**Figure 11:**
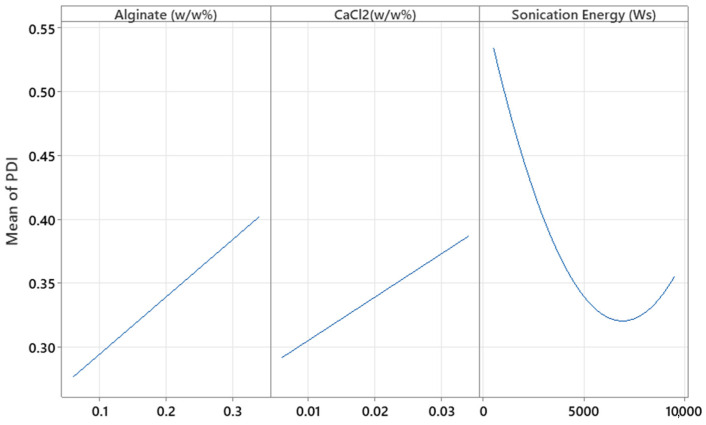
Plots of the main effects of alginate, CaCl_2,_ and sonication energy on the PDI.

Regarding PDI, different effects were observed. The levels of alginate, CaCl_2_, sonication energy, and their interaction were significant (*p* = 0.008, *p* = 0.035, *p* = 0.001, and *p* = 0.016, respectively). In this case, by increasing the alginate and CaCl_2_ concentration, the PDI increased (Figure 11). Although in the screening study increasing alginate and CaCl_2_ concentrations appeared to decrease the PDI values, the associated *p*-values (*p* = 0.110 and *p* = 0.091, respectively) were not statistically significant, and the model explained only 52.24% of the variability in this parameter (R^2^ = 0.5224). These results suggest that the screening study provided only a limited and preliminary insight into the behavior of the system.

In contrast, the optimization study revealed significant effects and interactions, showing that higher concentrations of polymer and cross-linker can increase particle heterogeneity. This may be due to the formation of overly dense or irregular networks, leading to aggregation or incomplete cross-linking. Additionally, the combined effect of higher sonication energy could intensify particle breakup in an uneven manner, further increasing polydispersity.

In the case of sonication energy (Ws), a quadratic relationship was obtained, and an interaction was found. A minimum polydispersity index (PDI) value was observed at an intermediate level of sonication energy, indicating the existence of an optimal energy input for particle size uniformity. At lower energies, insufficient dispersion likely leads to incomplete particle formation or aggregation, while excessively high energies may induce cavitation phenomena. These high-energy conditions generate intense shock waves and turbulence, which increase mass transfer and can cause both agglomeration and fragmentation of particles, ultimately resulting in a broader particle size distribution [34].

Considering the high prediction values from the obtained model, statistical optimization of the formulation was carried out to minimize the PDI value (as an indicator of a more stable system), within the design space limits. As shown in Table 9, to obtain the lowest PDI value, within the design space (0.228), it is necessary to use 0.10% alginate, 0.006% CaCl_2,_ and 6909.09 Ws of sonication energy. According to a screening study, a value of 40% for the sonication amplitude and 0.10% for the Eudragit^®^ E100 was established. Modeling particle size was not considered necessary, as all particle sizes obtained in the experimental space were suitable for biotin encapsulation. Moreover, since the sonication amplitude and Eudragit^®^ E100 concentration did not have a significant impact on PDI or particle size in the previous study, intermediate values for these variables were selected for the optimization phase.

In order to assess the predictability of the model, two independent placebo formulations were produced. The bias from the theoretical predictions is reported in Table 10. Although the predictable level of the model was low (0.51), all the obtained results were in the 95% confidence interval (CI), and the bias was <10%. Furthermore, the Z-Ave obtained was 1088 nm for Placebo 1 and 1063 nm for Placebo 2, and the Z-Pot was −37.4.

### 3.3. Physicochemical Characterization of Biotin-Loaded MPs

The encapsulation efficiency of the biotin-loaded microparticles was 90.50% ± 0.26, indicating a highly robust and reproducible encapsulation process.

In addition to encapsulation efficiency, the biotin content (*w*/*w*) of the final formulation was calculated theoretically. Considering an initial biotin concentration of 0.05% *w*/*w*, the resulting DL% was approximately 0.045%, confirming that the active was successfully incorporated within the microparticle matrix with minimal loss.

In this work, biotin content was expressed relative to the total mass of the final dispersion rather than as drug loading on a dry particle basis. This approach was selected because the microparticles were not lyophilized, and the study aimed to characterize the formulation under the conditions in which it will be handled and stored in subsequent applications. While dry basis determination can be useful for comparative purposes, reporting the content in the liquid dispersion provides a more relevant measure for liquid-based dosage forms and early-stage formulation screening. The relatively low biotin content selected in this study (0.045% *w*/*w* in the final dispersion) was determined by the solubility and stability constraints observed during preliminary trials. Attempts to encapsulate higher concentrations of biotin (0.10% and 0.25% *w*/*w*) resulted in precipitation during the particle formation stage, leading to heterogeneity and reduced reproducibility of the microparticles. Therefore, 0.05% *w*/*w* was identified as the maximum concentration that could be fully dissolved and stably incorporated under the selected formulation and processing conditions. In addition, this concentration avoided saturation of the release medium, which could otherwise bias the release kinetics analysis. These results suggest that future studies could investigate alternative solubilization or complexation strategies to enable higher loading levels without compromising particle stability.

Biotin-loaded microparticles (MPs) exhibited a smaller particle size compared to that of the empty MPs, measuring 633.7 ± 77.15 nm with a polydispersity index (PDI) of 0.260 ± 0.021. This reduction in size can be attributed to the electrostatic interactions between biotin and Eudragit^®^ E100 during particle formation.

Biotin, initially dissolved in 0.1 N NaOH, remains in its deprotonated form (−COO^−^) due to the high pH, which exceeds its pKa of 4.5. Conversely, Eudragit^®^ E100, dissolved in 0.1 N HCl, remains protonated (−NH_3_^+^) and positively charged. The electrostatic attraction between the negatively charged biotin and the positively charged Eudragit^®^ E100 enhances polymer matrix compaction by promoting ionic cross-linking, resulting in the formation of smaller microparticles [35]. The pKa values of both biotin (4.5) and Eudragit^®^ E100 (7–7.3) play a crucial role in ensuring the stability of these charge interactions under physiological pH conditions [36]. Similarly, sodium alginate (pKa around 3.5–4.0) deprotonates at higher pH levels, ensuring the presence of negatively charged groups that can interact electrostatically with positively charged entities like Eudragit^®^ E100, further reinforcing the stability and structure of the microparticles.

The zeta potential was −44.77 ± 1.06 mV, indicating high particle stability.

### 3.4. Transmission Electron Microscopy

**Figure 12:**
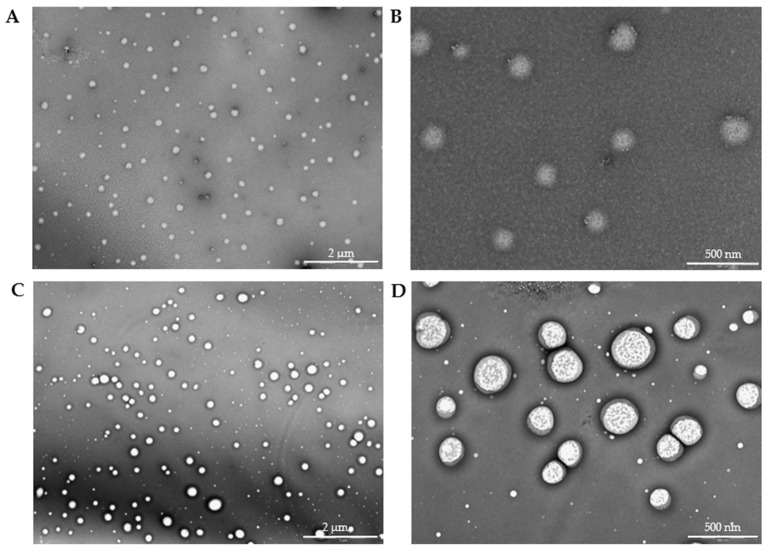
Transmission electron microscopy pictures of pta-stained microparticles with uranyl acetate. (**A**,**B**) alginate-MPs; (**C**,**D**) biotin-loaded alginate-MPs. Magnification of (**A**,**C**): 2 µm and (**B**,**D**): 500 nm.

The high resolution of the TEM images (Figure 12) allowed for the identification of the nucleus (brighter area) and its distinction from the polymeric framework (darker area). The spherical morphology was also confirmed. After analyzing 100 particles, the alginate-MPs exhibited a mean size of 136.36 ± 20.34 nm, while the biotin-loaded alginate-MPs, analyzed from 135 particles, had a mean size of 150.02 ± 38.19 nm. Moreover, Figure 12C,D shows that the particle size distribution was not homogeneous, in agreement with the PDI values obtained by DLS.

Notably, the TEM micrographs reveal a distinct core–shell morphology, particularly evident in the biotin-loaded microparticles. This structural feature corresponds well with the two-step fabrication process described in Section 2.7 [16,18,26,27,34,37]. In the first stage, alginate undergoes ionic gelation with CaCl_2_, forming a compact hydrogel core. In the second stage, the addition of Eudragit^®^ E100 followed by sonication promotes its deposition around the preformed alginate core. This results in the formation of a shell-like outer layer, driven by electrostatic interactions between the negatively charged alginate/Ca^2+^ matrix and the cationic groups of Eudragit^®^ E100.

The appearance of a brighter core and darker shell is consistent with this configuration, suggesting successful complexation between both polymers. This architecture has been associated with enhanced particle robustness, protection of the encapsulated compound, and modulation of release kinetics. Similar core–shell morphologies have been reported in other systems involving alginate and Eudragit^®^ polymers, supporting the observed structure and its functional relevance in this formulation strategy [14,38,39].

**Figure 13:**
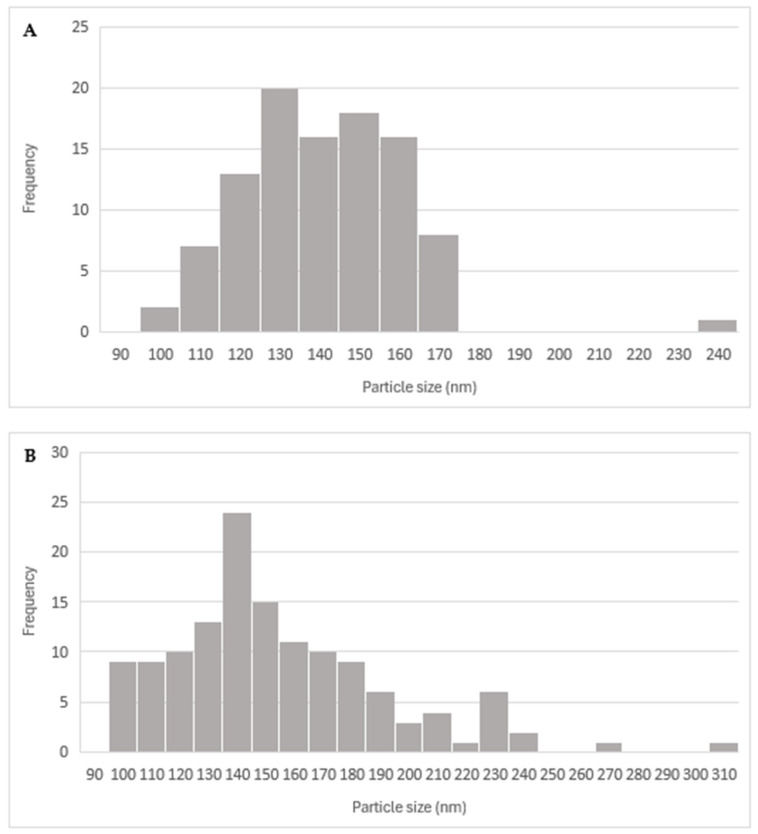
Histogram of (**A**) alginate-MPs from the measurement of the diameter of the particles (n = 100) and (**B**) biotin-loaded alginate-MPs from the measurement of the diameter of the particles (n = 135) using ImageJ (version 1.54g) software.

The diameter distribution of the particles, as measured by TEM, is presented in Figure 13 as a histogram.

The discrepancy between the particle sizes measured by DLS and TEM for both biotin-loaded and placebo microparticles can be explained by the fundamental differences between the two techniques [31,40,41,42,43].

In dynamic light scattering (DLS), the measurement reflects the hydrodynamic diameter, which includes not only the solid core of the particles but also any surrounding hydration layer and potential aggregates present in the dispersion [44]. This results in significantly larger values, as DLS is very sensitive to even a small number of larger aggregates, since light scattering intensity scales with the sixth power of the particle radius. In contrast, transmission electron microscopy (TEM) provides a direct “dry” image of the particles, measuring only the core size without the contribution of the solvation shell [45].

In our case, the biotin-loaded particles show a DLS size of 633 nm versus a TEM size of 150 nm. This indicates that in suspension, the biotin-loaded microparticles are either swollen, possess a significant hydration layer, or form loose aggregates, leading to an increased effective hydrodynamic diameter. On the other hand, the placebo microparticles have an even larger DLS size (800–1000 nm) compared to a TEM size of 136 nm. This suggests that the placebo formulation may have a higher tendency to aggregate or swell in solution, likely due to differences in surface properties or the absence of biotin, which could otherwise impart some steric or electrostatic stabilization.

Thus, the larger sizes observed by DLS are not contradictory but rather complementary to the TEM results: while TEM reveals the intrinsic particle dimensions, DLS reflects the dynamic behavior in solution and the influence of the surrounding medium. This interpretation aligns with previous studies [31,35], where DLS consistently reports higher hydrodynamic diameters due to these factors.

### 3.5. In Vitro Release Test

**Figure 14:**
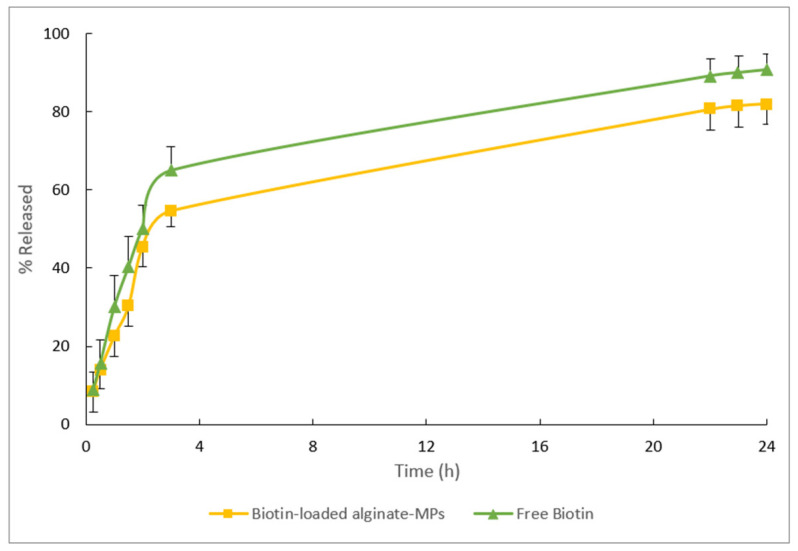
Mean release data obtained from the free biotin and biotin-loaded alginate-MPs after 24 h of the in vitro release test.

The release profiles of the free biotin and biotin-loaded MPs can be seen in Figure 14. After 24 h, 90% of the biotin formulated in an aqueous solution (free biotin) was released, and approximately 80% of the biotin encapsulated in alginate-MPs.

After adjusting the mean release data with the different mathematical models (see Table 11), it was observed that the Weibull model provided the lowest AIC values, indicating the best overall fit for both formulations.

For free biotin, the fitted Weibull parameter *β* was found to be 1.002. This is characteristic of first-order release kinetics, where the release rate is directly dependent on the concentration gradient. Such behavior is consistent with previous studies reporting that systems without additional release restrictions typically exhibit an exponential profile [46,47].

In contrast, biotin encapsulated in alginate microparticles yielded a *β* value of 0.62. According to Papadopoulou et al. [24] and supported by Monte Carlo simulation studies [48], *β* values below 0.69 suggest that the release mechanism is dominated by Fickian diffusion within a fractal or disordered medium. This indicates that the alginate matrix imposes a complex internal structure, which restricts and modulates drug diffusion, thereby altering the release profile. The electrostatic interaction between biotin and the polymers could explain the different release profiles.

These results can be further understood by considering the structural and electrostatic characteristics of the polymeric matrix. Upon ionic cross-linking with Ca^2+^, alginate forms a hydrogel network composed of guluronic acid-rich regions that create a dense, yet disordered structure with fractal and tortuous diffusion pathways [49,50]. These pathways physically restrict the movement of encapsulated molecules, which is consistent with the observed *β* value of 0.62 and the characteristics of Fickian diffusion in a heterogeneous medium.

Moreover, biotin’s anionic character (–COO^−^) under the formulation conditions (pH > pKa = 4.5) allows it to engage in ionic interactions not only with the cationic groups of Eudragit^®^ E100 (–NH_3_^+^) but also with calcium-cross-linked alginate chains. These electrostatic interactions promote additional ionic cross-linking and matrix compaction, leading to reduced porosity and diffusivity [51,52,53]. The combination of a physically constrained gel network and strong ionic interactions results in a more compact and stabilized polymeric architecture, thereby slowing biotin diffusion and shifting the release mechanism away from simple first-order kinetics toward a more regulated, matrix-controlled diffusion process.

The difference in *β* values further supports the role of the alginate matrix in shifting the release mechanism: while free biotin exhibits a release profile largely governed by the concentration gradient (first-order kinetics), the encapsulated system is controlled by diffusion through a heterogeneous, fractal-like matrix, as previously described by Ritger and Peppas [54] and Siepmann and Peppas [55].

As shown in Figure 14, 50% of the drug is released within three hours. Upon placement in the release medium, an initial large amount of drug is released before the release rate stabilizes, a phenomenon generally known as “burst release”. This burst release occurs over a very short period relative to the entire release process. Several studies have reported burst release but have not provided detailed explanations [56,57,58]. In this context, the burst effect is considered desirable, as it allows for immediate drug availability followed by controlled release levels. Drug release from hydrophilic matrices depends on the rate of matrix hydration and the matrix’s ability to form gels, both of which influence drug diffusion, gel formation, and erosion. Rapid hydration facilitates the formation of a gel layer, which can help limit excessive drug release during the initial phase. One explanation for the burst effect is that some drug may become trapped on the surface of the polymer matrix during manufacturing and is quickly released when exposed to the release medium. Additionally, the chemical and physical properties of the drug itself can significantly impact the burst effect in controlled release systems. The burst effect is more frequently observed with small molecular weight compounds, such as biotin [59,60].

In summary, these findings demonstrate that encapsulation in alginate microparticles modulates the biotin release mechanism—from an exponential, concentration gradient-driven release (*β* ≈ 1.002 in free biotin) to a diffusion-controlled release in a fractal medium (*β* = 0.62 in encapsulated biotin). These results not only confirm the suitability of the Weibull model for describing the complete release profile but also highlight the critical influence of the carrier’s internal structure on the release mechanism [30,48].

### 3.6. Scale-Up of Alginate-MPs

To enter the pharmaceutical market, specific requirements must be met, i.e., having affordable, large-scale production techniques along with concurrent compliance with regulatory standards. An easy production process running in a laboratory setup becomes useful only when it can be taken to a large scale. Large-scale processes require qualified lines to function effectively [61,62].

One of the important aspects to optimize in the design of microparticles as a delivery system is the control of particle size and polydispersity. Optimization becomes especially important when the formulation needs to be scaled up for industrial production [63,64]. To translate this formulation into large-scale production, we investigated two critical parameters and their correlation in the process. The manufacturing process was optimized to identify the best parameter combination in terms of the target response of Z-Ave and PDI. The parameters chosen include batch size and sonication cycles. Results of scale-up production are compiled in Table 12.

Surface response methodology was employed using Minitab software, with a significance level of α = 0.05 for the level terms, to evaluate the effects of the experimental variables. However, no statistically significant factor (*p* < 0.05) was found that could explain the change in the hydrodynamic diameter and the PDI. On a laboratory scale, the total energy applied influences the Z-average and PDI, with higher energy application leading to lower values for both parameters. For PDI, it was observed that at intermediate energy levels, the PDI was minimized compared to high energy levels. In these cases, the total energy applied per liter was consistently greater than that used on the laboratory scale. This discrepancy likely accounts for the observed differences. The energy utilized may have exceeded the necessary amount required to evaluate the impact of the studied variables effectively.

**Figure 15:**
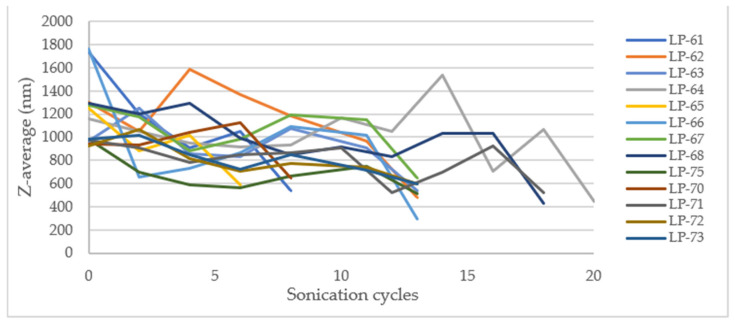
Effect of sonication cycles on the Z-average (nm).

**Figure 16:**
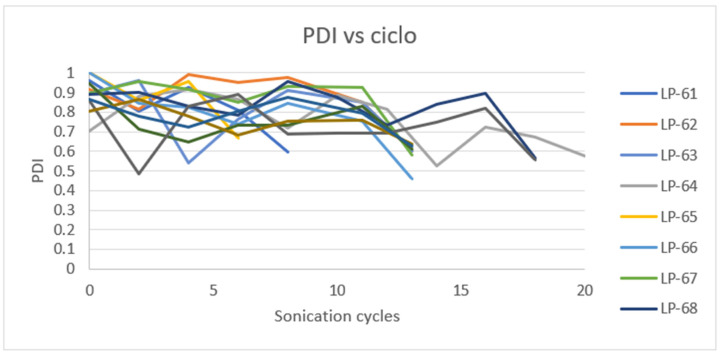
Effect of sonication cycles on the PDI.

According to laboratory-scale observations, a decrease in Z-Ave and PDI was anticipated with increased energy applied. However, this significant reduction only occurred after the addition of Eudragit^®^ E100 (Figure 15 and Figure 16).

The outcome may be due to the low percentage of CaCl_2_ used and the high energy applied, which might be insufficient for complete and effective cross-linking of the alginate, resulting in large and polydisperse microparticles (MPs). The microparticles obtained in the initial stage of the process exhibit high robustness, as they are not significantly influenced by the batch size or sonication cycles. This suggests that the first sonication stage after mixing all components can potentially be omitted, proceeding directly to the second stage (addition of Eudragit^®^ E100 and sonication). At the industrial manufacturing level, this modification could reduce production time and save energy, leading to a decrease in overall production costs.

The analysis examined the effects of sonication cycles and batch size on the PDI and hydrodynamic size, revealing that both variables significantly decreased after the addition of Eudragit^®^ E100 (stage 2). The average diameter and PDI were calculated before the addition of Eudragit^®^ E100 and compared with the values obtained afterward. The ratios of the resulting values (diameter and PDI after/diameter and PDI before addition), the average percentage of these ratios, and their standard deviation (SD) were computed. These parameters were not evaluated at the laboratory scale because, during the screening study, the concentration of Eudragit^®^ E100 did not significantly affect the variables studied. The results are summarized in the following table.

After calculating the ratio before and after the addition of Eudragit^®^ E100 to study the relationship between both variables, Table 13 indicates that for hydrodynamic diameter, there is a reduction of 54 ± 14%, suggesting that the particles formed after Eudragit^®^ E100 addition are nearly half the size. For PDI, the ratio before and after shows more similar values, at 70 ± 8%, indicating a reduction in PDI by approximately 30% due to the addition of Eudragit^®^ E100. These results suggest that the inclusion of Eudragit^®^ E100 is essential for the proper formation of microparticles and is an important factor in the process on a large scale [65].

Eudragit^®^ E100, as a cationic copolymer, coats the preformed MPs, forming a polyelectrolyte complex between its positively charged groups and the negative residues of the alginate-Ca MPs. This interaction results in more compact and rigid MPs, as indicated by the lower size and PDI values observed [44].

However, the effects of sonication cycles and batch size following Eudragit^®^ E100 addition have not been determined. A new experimental study with statistical analysis focusing on the second stage of the process may be required.

## 4. Conclusions

The study effectively demonstrated the formulation and optimization of biotin-loaded microparticles utilizing alginate, Eudragit^®^ E100, and CaCl_2_. The encapsulation of biotin in these microparticles presents several advantages. Their high encapsulation efficiency (%EE), favorable physicochemical properties at lab scale (size, low polydispersity, morphology, and Z-potential), and in vitro release profile render these microparticles an attractive solution for addressing biotin deficiencies. To the best of our knowledge, this is the first study reporting the encapsulation of biotin within alginate-based microparticles for controlled drug delivery applications. Although further studies are required to understand and enhance the scale-up process of these particles, the versatility and effectiveness of alginate microparticles in overcoming challenges associated with hydrophilic drug delivery remain highly promising. In addition, the findings support the broader use of polymer-based encapsulation systems as scalable and adaptable platforms for delivering other unstable or low-permeability active ingredients, expanding their potential in both pharmaceutical and functional product development.

## Figures and Tables

**Table 1:** Levels studied for Plackett–Burman design.

Factor	Lower Level	Higher Level
Alginate (% *w*/*w*)	0.10	0.30
Eudragit^®^ E100 (% *w*/*w*)	0.05	0.20
CaCl_2_ (% *w*/*w*)	0.01	0.03
% Sonication amplitude	20	60
Total work energy (Ws)	2000	8000

**Table 2:** Gradient profile for the HPLC analysis of biotin.

Time (min)	Mobile Phase A (%*v*/*v*)	Mobile Phase B (%*v*/*v*)
0	90	10
20	90	10
25	50	50
40	50	50
45	90	10
50	90	10

**Table 3:** Different kinetic models and equations were tested.

Kinetic Model	Equation
First-Order	$F=100\cdot ({1-e}^{\left(-k_{1}t\right)})$
Higuchi	$F={K_{H}\cdot \;t}^{1/2}$
Korsmeyer–Peppas	$F={K_{KP}\cdot \;t}^{n}$
Weibull	$F=100\times ({1-e}^{\left(-t^{\beta }/\alpha \right)})$

*F* is the fraction of active released at time *t*, *F_max_* is the maximum fraction of active released (i.e., at infinite time), *K*_1_ is the first-order constant, *K_H_* is the Higuchi constant, *K_KP_* is the Korsmeyer–Peppas constant (related to the structural and geometric character of the drug-release matrix), *n* is the diffusional exponent indicating the drug-release mechanism (if *n* is less than 0.43, then a Fickian diffusion release mechanism is implied; if *n* is between 0.43 and 0.85, then the release mechanism follows an anomalous transport mechanism), T_d_ represents the time at which 63.2% of the drug is released, and *β* is the Weibull shape parameter. For values of *β* lower than 0.75, the release follows a Fickian diffusion, either in Euclidean (0.69 < *β* < 0.75) or fractal (*β* < 0.69) space. Values of *β* in the range of 0.75–1.0 indicate a combined mechanism, which is frequently encountered in release studies [24,25].

**Table 4:** Levels studied for central composite design of scale-up.

Factor	Lower Level	Higher Level
Sonication cycles	6	20
Batch size (L)	0.775	5.725

**Table 5:** Influence of the excipients’ proportions and fabrication parameters on the physicochemical characteristics of MPs.

Batch	Alginate (%*w*/*w*)	CaCl_2_ (%*w*/*w*)	Eudragit^®^ (%*w*/*w*)	Amplitude (%)	Sonication Energy (Ws)	Z-Ave (nm)	PDI	ZP (mV)
LP-01	0.3	0.01	0.20	60	8000	1040	0.350	−48.5
LP-02	0.3	0.01	0.20	60	2000	1599	0.419	−43.1
LP-03	0.3	0.03	0.20	20	8000	1950	0.516	−47.1
LP-04	0.3	0.03	0.05	20	2000	3305	0.243	−60.6
LP-05	0.3	0.03	0.05	60	2000	2769	0.039	−56.4
LP-06	0.1	0.01	0.05	20	2000	557.3	0.534	−48.2
LP-07	0.1	0.03	0.20	60	2000	620.3	0.379	−26.2
LP-08	0.1	0.01	0.20	20	2000	1133	0.752	−25.9
LP-09	0.1	0.01	0.05	60	8000	339.7	0.628	−42.7
LP-10	0.3	0.01	0.05	20	8000	1365	0.369	−54.3
LP-11	0.1	0.03	0.05	60	8000	255.1	0.481	−43.6
LP-12	0.1	0.03	0.20	20	8000	664.5	0.237	−26.7

**Table 6:** Screening model equations for monitoring the effect of the alginate, CaCl_2,_ and Eudragit^®^ E100 concentrations and energy of sonication on the physicochemical characteristics of the MPs.

Quadratic Polynomial Model Equation	R^2^	Adj R^2^
Y_1_ = −91 + 7048X_1_ + 2916X_2_ − 0.1214X_3_	0.8405	0.7807
Y_2_ = 0.784 − 0.896X_1_ − 9.64X_2_	0.5224	0.4163
Y_3_ = −39.76 − 80.58X_1_ + 98.1X_4_	0.9355	0.9212

X_1_: concentration (%*w*/*w*) of alginate; X_2_: concentration (%*w*/*w*) of CaCl_2_; X_3_: sonication energy (Ws); X_4_: concentration (%*w*/*w*) of Eudragit^®^ E100; Y_1_: hydrodynamic size; Y_2_: polydispersity index; Y_3_: zeta potential (mV).

**Table 7:** Influence of alginate, CaCl_2,_ and energy on the Z-Ave and PDI parameters.

Batch	% Alginate (*w*/*w*)	% CaCl_2_ (*w*/*w*)	Total Work Energy (Ws)	Z-Ave (nm)	PDI
LP-13	0.20	0.020	9200	759	0.386
LP-14	0.10	0.030	2000	1144	0.488
LP-15	0.20	0.020	5000	928	0.330
LP-16	0.06	0.020	5000	679	0.323
LP-17	0.20	0.020	5000	1408	0.284
LP-18	0.34	0.020	5000	1786	0.422
LP-19	0.30	0.030	2000	1678	0.479
LP-20	0.30	0.010	2000	1780	0.462
LP-21	0.20	0.020	5000	1251	0.351
LP-22	0.10	0.010	2000	770	0.305
LP-23	0.30	0.010	8000	1173	0.306
LP-24	0.20	0.034	5000	900	0.336
LP-25	0.20	0.006	5000	1327	0.293
LP-26	0.20	0.020	5000	1428	0.361
LP-27	0.10	0.030	8000	569	0.255
LP-28	0.20	0.020	5000	1252	0.448
LP-29	0.10	0.010	8000	833	0.240
LP-30	0.30	0.030	8000	2035	0.436
LP-31	0.20	0.020	800	1477	0.537
LP-32	0.20	0.020	5000	1276	0.303

**Table 8:** Response surface model equation for monitoring the effect of alginate and CaCl_2_ concentration and energy on the properties of microparticles.

Quadratic Polynomial Model Equation	R^2^	Adj R^2^
Y_1_ = 648 + 4111X_1_ − 0.0494X_3_	0.7255	0.6910
Y_2_ = 0.4111 + 0.448X_1_ + 3.40X_2_ − 0.000072X_3_ + 0.0000002 X_3_ * X_3_	0.7196	0.6449

X_1_: concentration (%*w*/*w*) of alginate; X_2_: concentration (%*w*/*w*) of CaCl_2_; X_3_: sonication energy (Ws); Y_1_: hydrodynamic diameter; Y_2_: polydispersity index.

**Table 9:** Prediction of variables for alginate particles with minimum PDI value.

Alginate (*w*/*w*%)	CaCl_2_ (*w*/*w*%)	Sonication Energy (Ws)	PDI Fit	Composite Desirability
0.10	0.006	6909.09	0.228	1.00

**Table 10:** Results after characterization of the final formulation with bias with respect to the theoretical value.

Property	Theoretical Value (95% CI)	Placebo Formulation 1	Placebo Formulation 2	Bias (%)
PDI	0.228 (0.103; 0.359)	0.245	0.255	9.65%

**Table 11:** Model selection and parameter estimation of the free biotin and biotin-loaded alginate-MPs. Bold type indicates the selected model, based on the lowest AIC.

Formulation	Model	AIC	Parameters	Value
Free Biotin	First-order	71.33	$k\;(h^{-1})$	0.1546
	Higuchi	69.75	$k_{H}\;({\%h}^{-1/2})$	20.38
	Korsmeyer–Peppas	69.26	$k_{KP}\;{(\%h}^{-n})$	39.08
			n	0.2246
	**Weibull**	**55.62**	**α**	**3.037**
			β	**1.002**
Biotin-loaded Alginate-MPs	First-order	71.03	$k\;(h^{-1})$	0.1128
	Higuchi	65.15	$k_{H}\;({\%h}^{-1/2})$	18.18
	Korsmeyer–Peppas	66.39	$k_{KP}\;{(\%h}^{-n})$	22.33
			n	0.4578
	**Weibull**	**57.04**	**α**	**3.723**
			β	**0.6248**

**Table 12:** Scale-up production of alginate-MPs results.

Batch	Sonication Cycles	Total Real Work Energy (Ws)	Batch Size (L)	Z-Ave (nm)	PDI
LP-61	8	27,857	1.500	538.2	0.599
LP-62	13	84,708	3.250	479.6	0.604
LP-63	13	90,488	3.250	540.4	0.601
LP-64	20	130,340	3.250	446.5	0.577
LP-65	6	41,887	3.250	590.2	0.668
LP-66	13	87,935	3.250	490.7	0.462
LP-67	13	20,801	0.775	648.7	0.581
LP-68	18	55,634	1.500	424.8	0.567
LP-69	13	151,890	5.725	513.8	0.612
LP-70	8	84,592	5.000	650.9	0.586
LP-71	18	195,750	5.000	524.6	0.555
LP-72	13	89,178	3.250	597.7	0.638
LP-73	13	95,669	3.250	597.9	0.627

**Table 13:** Effect on Z-Ave and PDI before and after adding Eudragit^®^ E100.

Batch	Z-Ave (nm) Before	Z-Ave (nm) After	Ratio	PDI Before	PDI After	Ratio
LP-61	1226	538	0.44	0.874	0.599	0.69
LP-62	1242	480	0.39	0.917	0.604	0.60
LP-63	982	540	0.55	0.820	0.601	0.73
LP-64	1055	447	0.42	0.771	0.577	0.75
LP-65	1049	590	0.56	0.941	0.668	0.71
LP-66	1021	491	0.29	0.835	0.462	0.55
LP-67	1112	649	0.58	0.913	0.581	0.64
LP-68	1050	425	0.41	0.856	0.567	0.66
LP-69	706	514	0.72	0.769	0.612	0.80
LP-70	1011	651	0.64	0.913	0.586	0.64
LP-71	824	525	0.64	0.745	0.555	0.75
LP-72	837	598	0.71	0.775	0.638	0.82
LP-73	856	598	0.70	0.808	0.627	0.78
	Mean ratio ± SD (%)		54 ± 14%	Mean ratio ± SD (%)		70 ± 8%

## Data Availability

Data is available upon request due to intellectual property.
